# Plastic pollution amplified by a warming climate

**DOI:** 10.1038/s41467-024-46127-9

**Published:** 2024-03-06

**Authors:** Xin-Feng Wei, Wei Yang, Mikael S. Hedenqvist

**Affiliations:** 1 Department of Fibre and Polymer Technology, KTH Royal Institute of Technology, SE–100 44 Stockholm, Sweden; 2 College of Polymer Science and Engineering, Sichuan University, 610065 Chengdu, PR China

**Keywords:** Environmental impact, Environmental chemistry, Climate-change impacts

## Abstract

Climate change and plastic pollution are interconnected global challenges. Rising temperatures and moisture alter plastic characteristics, contributing to waste, microplastic generation, and release of hazardous substances. Urgent attention is essential to comprehend and address these climate-driven effects and their consequences.

Earth’s global average temperature has increased by approximately 1 °C above pre-industrial levels with a current rate of ca. 0.2 °C per decade, primarily due to huge greenhouse gas emissions^1^. The Paris Agreement’s target of limiting global warming to 1.5 °C is projected to be breached in the near term^2^. Extreme regional heatwaves are also showing immediate and marked temperature spikes, sometimes exceeding 10 °C above normal levels^3^. In 2022, extreme heatwaves led to temperature records in many regions (e.g., 40.3 °C in the United Kingdom and 49.1 °C at Smara (Morocco))^4^. In 2023, the trend continued with July being the hottest month ever recorded^3^. The frequency, intensity, and duration of heatwaves have all increased^5^. In Phoenix, Arizona, during July 2023, all days except one, exhibited a maximum temperature exceeding 110 °F (43 °C)^3^. The high temperatures have caused severe impacts on ecosystems and societies, including excess mortality, wildfires, and harvest failures^4^. This will get even worse in the future as heatwaves are projected to be more intense, frequent, and prolonged due to the enhanced global warming^5^, and developing El Niño conditions^6,7^. In addition, a warmer atmosphere increases the evaporation of moisture and, with each 1 °C rise in temperature, saturated air can hold 7% more water vapor^8^. The average moisture content of the atmosphere has increased by approximately 4% since the 1970s^8^.

## Deteriorated properties and increased waste

Polymer materials, mainly plastics and rubbers, are notably sensitive to temperature and moisture fluctuations. As temperatures rise, polymers undergo thermal expansion, leading to inferior properties^9^. Commonly used plastics like polyethylene, polypropylene, and polyvinyl chloride can experience an over 20% decrease in stiffness with a service temperature rise from 23/24 to 40 °C^10,11^. Time-dependent changes in mechanical properties, such as creep (slow deformation process of materials under constant or varying load), and stress relaxation (the decrease in stress response under sustained deformation), will also accelerate. Furthermore, rising temperatures negatively affect other important properties, such as gas and water vapor barrier properties in food packaging, essential for food preservation. For example, ethylene vinyl alcohol, a common gas barrier polymer, can experience a reduction of over 75 % in oxygen barrier efficiency as the temperature increases from 23 to 40 °C^12^, potentially leading to food spoilage.

In addition to these immediate effects, a warming climate speeds up long-term property loss due to accelerated ageing^9^. Polymers degrade/age over time from factors like heat, light, moisture, chemicals, and mechanical stress, involving oxidation, UV degradation, hydrolysis, biodegradation, and additive migration^9,13^. Temperature is a key factor in all these processes. According to the Arrhenius law, the degradation rate increases exponentially with increasing temperature – with a typical activation energy of 50 kJ/mol for plastic degradation, every 10-degree temperature rise doubles the degradation rate^13^.

For hygroscopic polymers, such as thermoplastic starch and other biopolymers, polyamides, and polyesters, moist conditions can add to the negative effects of rising temperature. Water is a powerful “plasticizer” in systems where the uptake is sizeable, leading to a softer and weaker material. Water uptake may also increase the creep rate and the risk of degradation through hydrolysis.

A warmer climate therefore exposes polymers to more challenging conditions, resulting in the deterioration of plastic properties in both the short and long terms. This leads to more frequent failures of plastic components and products, resulting in reduced durability and shorter service life. Consequently, failed products often need to be replaced, increasing the generation of plastic waste and exacerbating the problem of plastic pollution. Extensively degraded plastic waste is generally unsuitable for traditional recycling due to property loss, increasing the likelihood of such waste being excluded from current plastic waste management systems and ending up in both terrestrial and aquatic environments.

### Escalated leaching risk of plastic-associated chemicals

Over 13,000 chemicals are associated with plastics and their production, and among them over 3,200 have been identified as potential concerns due to their hazardous properties^14^. These chemicals consist of residual monomers/oligomers from the polymerization process, compounds formed during polymer degradation, and a wide range of additives like lubricants, flame retardants, plasticizers, antioxidants, colorants, and UV/heat stabilizers^14^. These hazardous chemicals can be emitted and released throughout the plastic lifecycle, posing risks to ecosystems and humans. As temperatures rise, both the diffusion and evaporation rates of the species accelerate, intensifying the leaching of these substances into the air, soil, and water^15^. In addition, the accelerated ageing processes in a warmer climate result in faster production of hazardous degradation products^16^. This amplifies the risk of plastic-associated chemicals entering our ecosystems. As a common example, temperature significantly influences the emission of volatile organic compounds (VOCs) from automobile interior plastic and elastomer components, potentially causing ‘sick car syndrome’^14^. In the case of hygroscopic polymers, the combination of high temperatures and high relative humidity may exacerbate the release of chemicals further.

### Increased microplastic risk

Another concern regarding plastic pollution is the formation of microplastics (tiny particles under 5 mm), due to their persistence, wide distribution, and adverse effects. They originate from the manufacturing of plastics (primary sources) and the gradual degradation of plastic items (secondary sources)^17^. A warmer climate accelerates polymer degradation^9^ and thus the breakdown of plastic items into smaller species, substantially expediting the generation of secondary microplastics. Accelerated ageing yields microplastics with a greater degree of degradation, which can increase their toxicity due to the accumulation of degradation products in the microplastic particles. The ageing process profoundly alters the physicochemical properties of these microplastics, subsequently affecting their environmental behaviors^16^. These changes encompass surface charge, biofilm formation, transportation, adsorption behaviors, and interactions with their surroundings^16^. For instance, as microplastics age, their surface roughness tends to increase and their hydrophobicity decreases. These changes make them more conducive to bacterial colonization and the subsequent formation of biofilms^16^. Therefore, the acceleration of plastic degradation, induced by a warmer climate, not only increases the rate at which microplastics are generated but also enhances the ecotoxicity of the formed microplastic particles. This further exacerbates the issue of microplastic pollution and poses long-lasting risks to living organisms in both terrestrial and aquatic environments. In aquatic environments, the rising water temperatures, often due to marine heatwaves and global warming, also hasten the degradation of plastic litter and the subsequent release of microplastics. Note that microplastics also experience accelerated ageing in a warming climate, which leads to quicker fragmentation into nanoplastics and their eventual disintegration. This implies that plastics have a reduced persistence in environments under conditions of climate warming.

### Increased demands for plastics

Climate change may also significantly increase the demand for materials with the properties of plastics in various applications. With rising temperatures, the need for electrical appliances, such as air conditioners, fans, and refrigerators, all of which heavily rely on plastic components, escalates, as observed in Europe during hot summers^18^. Additionally, initiatives such as renewable energy projects, electrification of transportation, and climate-resilient infrastructure require a significant number of plastic components. Intensified climate-related disasters like wildfires, floods, hurricanes, cyclones, and typhoons also contribute to plastic demand as they require plastics for reconstruction, emergency shelters, personal protective equipment (PPE), and humanitarian aid supplies. These disasters, unfortunately, lead to the widespread destruction of plastics in use, converting them into waste within the affected area on a massive scale. This heightened demand for plastics leads to increased production, consumption, and subsequent waste generation, exacerbating the issue of plastic pollution. Thus, carefully managing plastic use in climate projects is crucial, ensuring our efforts are both environmentally effective and sustainable in material use.

### A vicious circle

**Fig. 1:**
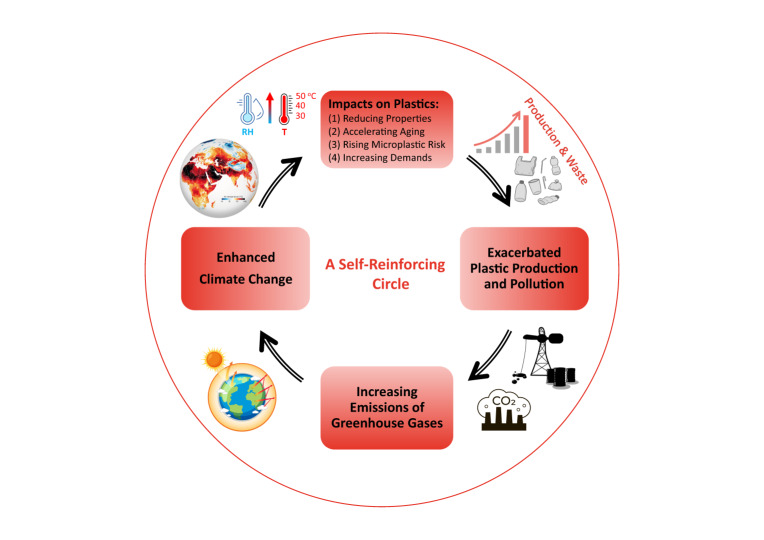
A self-reinforcing circle between climate change and plastic pollution. The map in the upper left corner represents the air temperatures in the Eastern Hemisphere 13th of July, 2022 (Source: NASA Earth Observatory, https://earthobservatory.nasa.gov/images/150083/heatwaves-and-firesscorch-europe-africa-and-asia).

To conclude, a warming climate has consequences for the use, ageing, and disposal of plastics, fueling plastic pollution with more waste generation, increased release of chemicals from plastics, and generation of more microplastics. On the other hand, the plastic industry is widely known as a significant contributor to emissions of greenhouse gases and, consequently, climate change^19^. This creates a paradoxical situation where the changing climate drives the demand for plastic, further contributing to plastic pollution, while at the same time, the increasing production of plastics and elastomers exacerbates climate change. Thus, a self-reinforcing cycle is formed, creating a vicious circle between climate change and plastic pollution (Fig. 1).

Despite the significant role of climate change in intensifying plastic pollution^20^, this particular impact remains underemphasized. As global warming and heatwaves intensify, and with plastic production, usage, and waste reaching unprecedented levels, it is imperative that we urgently draw attention and mobilize efforts across all sectors involved in the plastic lifecycle. This encompasses the plastics manufacturing industry, sectors utilizing these materials such as electronics, construction, and food packaging, retailers, consumers, regulatory authorities, governments, environmental organizations, waste management services, and the academic and research community in both the plastics and environmental fields. Such collaboration is essential to enhance our understanding of how climate change affects plastic properties and pollution, both immediately and in the long term.

To effectively tackle the intertwined challenges of plastic pollution and climate change, we need a multi-dimensional strategy that encompasses global policy and regulation, technological advances, improved waste management, public engagement, and international collaboration. This approach should emphasize sustainable practices, economic incentives, community participation, and continual research to reduce environmental impacts effectively. For example, implementing a ban on single-use plastics, advocating for a circular economy through enhanced reuse and recycling of plastic items, and transitioning to alternative materials with lower carbon footprints and diminished environmental impacts, such as certain bio-based or biodegradable options, are crucial measures. These steps are critical in disrupting the vicious cycle of plastic pollution and climate change, addressing both issues collaboratively, and reducing their economic and environmental toll, ultimately leading to a more sustainable and resilient future.
