# Publisher Correction: Plastic pollution amplified by a warming climate

**DOI:** 10.1038/s41467-024-46860-1

**Published:** 2024-03-21

**Authors:** Xin-Feng Wei, Wei Yang, Mikael S. Hedenqvist

**Affiliations:** 1 Department of Fibre and Polymer Technology, KTH Royal Institute of Technology, SE–100 44 Stockholm, Sweden; 2 College of Polymer Science and Engineering, Sichuan University, 610065 Chengdu, PR China

**Keywords:** Environmental impact, Environmental chemistry, Climate-change impacts

Correction to: *Nature Communications* 10.1038/s41467-024-46127-9, published online 6 March 2024

In the original PDF of this article, the author name Xin-Feng Wei was incorrectly written as Wei. The original article PDF has been corrected. The HTML version was unaffected.

